# ﻿MycoPins: a metabarcoding-based method to monitor fungal colonization of fine woody debris

**DOI:** 10.3897/mycokeys.96.101033

**Published:** 2023-03-21

**Authors:** Maria Shumskaya, Nicholas Lorusso, Urvi Patel, Madison Leigh, Panu Somervuo, Dmitry Schigel

**Affiliations:** 1 Department of Biology, Kean University, Union, USA Kean University Union United States of America; 2 University of North Texas at Dallas, Dallas, USA University of North Texas at Dallas Dallas United States of America; 3 Biological and Environmental Sciences, University of Helsinki, Helsinki, Finland University of Helsinki Helsinki Finland

**Keywords:** Dead wood, metabarcoding, mycobiome, saproxylic fungi

## Abstract

The MycoPins method described here is a rapid and affordable protocol to monitor early colonization events in communities of wood-inhabiting fungi in fine woody debris. It includes easy to implement field sampling techniques and sample processing, followed by data processing, and analysis of the development of early dead wood fungal communities. The method is based on fieldwork from a time series experiment on standard sterilized colonization targets followed by the metabarcoding analysis and automated molecular identification of species. This new monitoring method through its simplicity, moderate costs, and scalability paves a way for a broader and scalable project pipeline. MycoPins establishes a standard routine for research stations or regularly visited field sites for monitoring of fungal colonization of woody substrates. The routine uses widely available consumables and therefore presents a unifying method for monitoring of fungi of this type.

## ﻿Introduction

Biological communities are formed at intersections of species niches and local environment, with assembly history strongly influencing overall community structure (Fukami et al. 2010). Early colonization events play a key role in establishment of pioneer communities, with species at these seminal stages competing for habitats and resources which are limited both spatially and temporally. An example of a habitat limited in both space and metabolic resources, and, at the same time, relatively slow in community turnover rates, is dead wood (Stokland et al. 2012). Saproxylic fungi are the major wood decaying organisms able to digest lignin and cellulose that would undergo community assembly as woody tissues undergo decomposition (Baldrian 2017). These fungal taxa form competitive meta-communities and coexist both in space and for the time of decaying (Abrego et al. 2014). Wood-decaying fungi have been shown to be latently present in angiosperms (Boddy et al. 2007; Parfitt et al. 2010), but for conifers the necessary information on structure and functioning of Basidiomycete and Ascomycete communities is limited (Boddy 2001; Küffer and Senn-Irlet 2005). The information on ecology of saproxylic fungi is essential for evaluating environmental threats to key fungal species, conservation, and management policies (Hawksworth 1991; Mueller and Schmit 2007; Allen and Lendemer 2015; Seibold et al. 2015; Moose et al. 2019). For example, the loss of dead wood has posed a threat to a quarter of all forest species in Finland (Siitonen 2001; Rassi et al. 2010).

While lignicolous Basidiomycota are taxonomically well-known (Niemelä 2005; Kotiranta et al. 2009) most ecological studies of this group have been based on fruit body survey data (Rayner and Boddy 1988; Boddy et al. 2007; Halme 2010). Recent advances in DNA sequencing and metabarcoding allow the analysis of cryptic fungi via extracting DNA from substrates such as soil or wood where the fungi are found but not identifiable using fruiting body morphology. Fungal metabarcoding is widely used in mycorrhiza mycology (Bruns and Kennedy 2009; Öpik et al. 2009; Tedersoo et al. 2009), but most molecular studies on fungi in dead wood focus on coarse woody debris (Kubartova et al. 2009; Gherbawy and Voigt 2010; Arnstadt et al. 2016; Moll et al. 2021). Fungal ecology of fine woody debris remains largely neglected with a few exceptions (Norden et al. 2004; Bassler et al. 2010; Juutilainen et al. 2011; Juutilainen et al. 2014; Krah et al. 2018; Brabcova et al. 2022) and this gap presents an opportunity to leverage metabarcoding to identify cryptic taxa.

**Figure 1.**
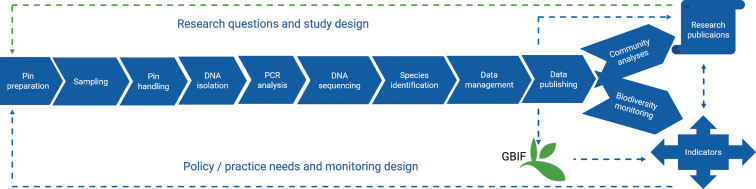
Scheme of the MycoPins method pipeline.

The niche boundaries and ecological processes governing community assembly of saproxylic species are important for shaping trajectories for dead wood decomposition (Fukasawa 2018), but a standard workflow for sampling these species is still lacking. Here, we describe a straightforward method for studying and monitoring dead wood colonization by fungi to fill this gap in knowledge and allow for consistency regardless of sampling location or other aspects of study design. The method combines standardized fieldwork protocol that includes experimental exposure of wooden furniture pins to lignicolous fungi in the environment, followed by subsequent metabarcoding analysis of fungal DNA from the colonized pins (Fig. 1). This standardized pipeline utilizes previously established metabarcoding methods, and we suggest that together with the sampling protocol the clear baseline will reduce difficulty in comparing studies that employ widely different designs.

Furniture pins are widely available and can be ordered from wood suppliers to match desired local timber species. The selection of pins appropriate for the local forest community through coordination of timber with local wood suppliers allows for tailoring of the method to account for issues such as the interrelationship between host and fungal community composition, e.g. (Balint et al. 2013). Transporting dry construction wood pins acquired prior to fieldwork should also be likely not problematic in terms of travelling to study locations that may involve crossing state or governmental borders. In the work presented here, we evaluate this method to explore its research potential and reliability in a hope to trigger higher resolution worldwide monitoring of fungal wood colonization. Using autoclaved (industry clean) pins, we also compared the effect of preservation method (dry *vs.* frozen) on the pins exposed to the fungal colonization in the environment.

We hypothesized that the pins placed in the soil would be colonized by decaying fungi (hypothesis 1). The null hypothesis states that the pins would not be colonized by fungi. Within the hypothesis 1, there are three options available: 1a – the pins would be colonized by saproxylic fungi with the communities undergoing succession during the decomposition, 1b – the pins would be colonized by soil mycorrhizal fungi, 1c – the fungi would be colonized by both groups of fungi. Our hypothesis 2 is that storage and handling methods would not affect the results of the experiment. The null hypothesis states that drying and freezing methods would preserve a different number of species.

## ﻿Materials and methods

### ﻿Field experiment

**Figure 2.**
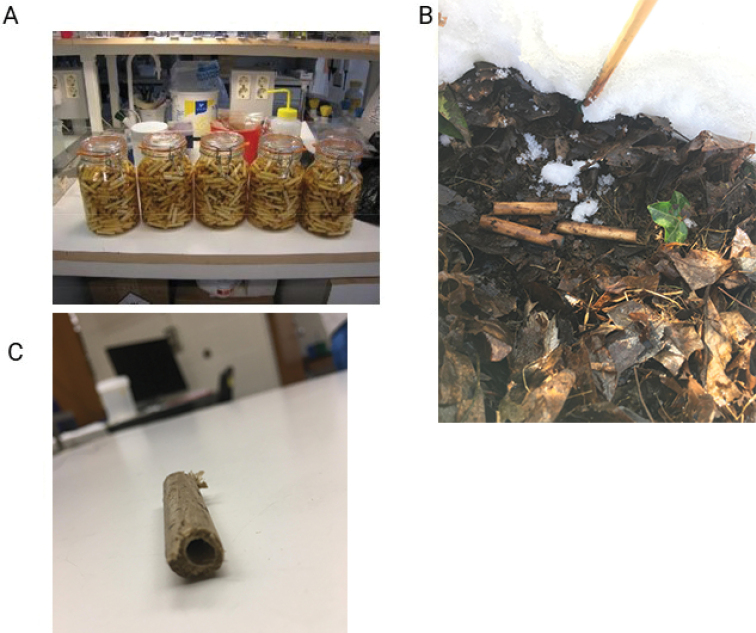
Preparation of pins for further analysis **A** sterilized pins **B** pin placement in soil **C** the interior of the colonized pins was extracted via drilling. Saw dust then was used to extract DNA.

A poplar pin 1 cm in diameter was purchased at a local hardware store in New Jersey, USA, and segmented into 3 cm long pins. The pins were then placed in a sterilizing jar and autoclaved (121 °C, 50 min). After sterilization, the pins were positioned in sets of three next to each other (Fig. 2), on November 23^rd^, 2020 in soil 2 cm from the surface, covered with debris and allowed to decay while being monitored for a set number of days (Table 1). Triplicates were placed 1 m from each other along one transect. Pins were placed in an urbanized wooded area in central New Jersey, USA. Upon extraction from soil, two pins for each triplicate were wrapped in brown paper and dried for 5 hours at 45 °C in a conventional food dehydrator, and one pin was frozen at -80 °C. Two sterile negative control pins were exposed to air in the field for 30 min and then one was dried and one frozen using the same methods used to store sample pins.

**Table 1.** Sample list for each pin (=sample), time from placement, and storage methods used.

Sample number	Date of extraction	Days decaying	Storage
0	–	0. Sterilized pins.	1: dried, 1: frozen at -80 °C
1	December 07, 2020	14	2: dried, 1: frozen at -80 °C
2	December 21, 2020	28	2: dried, 1: frozen at -80 °C
3	January 04, 2021	42	2: dried, 1: frozen at -80 °C
4	February 08, 2021	77	2: dried, 1: frozen at -80 °C
5	May 02, 2021	160	2: dried, 1: frozen at -80 °C

### ﻿DNA extraction

The interior of each pin was drilled by a 2 mm fire-sterilized drill bit and the sawdust was collected into sterile plastic centrifuge tubes. DNA was isolated using PowerSoil DNA isolation kit (Qiagen, MD, USA) according to manufacturer’s instructions. For the homogenization of the cell lysis solution with the saw dust, BeadBug (Benchmark Scientific, NJ, USA) homogenizer was utilized. Concentrations of extracted DNA for each sample were measured using NanoDrop spectrophotometer (ThermoFisher Scientific, USA) (absorbance at 260 nm). Extracted DNA was stored in 10 mM Tris buffer pH 8.0 at -80 °C.

### ﻿PCR

PCR for the ITS2 gene region from the extracted DNA was carried out as in (Clemmensen et al. 2016). The primers used were tagged versions of fITS7 forward (F), 5’-GTGARTCATCGAATCTTTG, and ITS4 as reverse (R), 5’-TCCTCCGCTTATTGATATGC (White et al. 1990; Ihrmark et al. 2012). To each primer, an individual 10-nucleotide sequence (tag) was added. Altogether, 10 pairs of primers were used which allowed to perform ITS2 amplification for 10 different samples and then mix them in one multiplex to be analyzed later by high-throughput sequencing. The list of resultant primers is presented in Suppl. material 1: table S1. 100 ng of extracted DNA from each individual pin was amplified with PCR using a primer pair with an individual tag (such as pair #1, #2 etc) for each DNA sample. PCR amplification was performed using Phusion High-Fidelity Master Mix (Thermo Fisher Scientific, MA, USA). The mix is supplied as X2 concentrate, and a typical PCR reaction of 50 µl consisted of 25 µl of the PCR mix, 1 µl of each 5 µM primer, DNA to make a final amount of 100 ng in a PCR reaction tube, and DNAse-free water to make up the volume to 50 µl. The PCR conditions were 5 min at 94 °C, 30 cycles of: 30 s at 94 °C, 30 s at 52 °C and 30 s 72 °C, and the final step of 5 min at 72 °C. While we provided our PCR conditions, they can be modified depending on the PCR mix or Taq-polymerase available at the research laboratory.

For a negative control of PCR and subsequent sequencing and data analysis, DNAse-free water was used in place of the DNA. The reaction was set with a pair of primers with an individual tag, and the reaction was processed the same way as all other samples. Since no PCR product was detected in this control, all volume of the negative control reaction was used for the subsequent purification and sequencing steps. For a positive control, a SynMock plasmid collection provided by Drs. J. Palmer and D. Lindner, USDA Forest services (Palmer et al. 2018) was used. SynMock collection consists of a mix of 11 pUC57 bacterial plasmids each containing an insert of an individual artificially synthesized fungal ITS region. The ITS sequences were generated by Lindner laboratory and are available in the OSF repository (https://osf.io/4xd9r/, (Palmer et al. 2018). Amplification of this artificial “fungal community” was processed as a separate sample with its own tag. The resultant amplicon was added to the mix of the amplicons of investigated communities, which serves as a control to both PCR amplification, high-throughput sequencing and data analysis. This SynMock plasmid mix was prepared with a concentration of 0.1ng/ul of each plasmid in a mix, and then amplified with a pair of primers with an individual tag, mixed with the rest of the samples and processed the same way as all other samples. After the high-throughput sequencing analysis and bioinformatics analysis, the SynMock “species” were detected in amounts correlating with the added plasmid concentration and were detected only in the amplicons with the tagged primers used for its preparation. This way, we were able to confirm the validity of the method.

The PCR products were then visualized for successful PCR confirmation by gel electrophoresis (see an example in Suppl. material 1: fig. S1). Mag-Bind RXN Pure Plus Quick kits (Omega Biotek, GA, USA) were used to purify the PCR products according to the manufacturer’s standard protocol. Concentrations of the purified tagged amplicons were measured using Qubit 3.0 fluorimeter using dsDNA HS Assay kit (ThermoFisher Scientific, MA, USA).

### ﻿High-throughput sequencing

Purified tagged PCR amplicons were pooled in a multiplex in equal 100 ng amounts, then the pooled sample was concentrated using Amicon Ultra-0.5 30K centrifugal filters (Millipore Sigma) to the volume and concentration required by the sequencing facility (at least 20 µl, 20 ng/µl). DNA library preparations, sequencing reactions, and adapter sequences trimming were conducted by Genewiz (now Azenta, South Plainfield, NJ, USA), using their Amplicon EZ service (provides approximately 50 000 reads per sample). DNA library preparation was performed using NEBNext Ultra DNA Library Prep kit following the manufacturer’s recommended procedure (Illumina, San Diego, CA, USA). In short, end repaired adapters were ligated after adenylation of the 3’ends followed by enrichment by limited cycle PCR. DNA libraries were validated on the Agilent TapeStation (Agilent Technologies, Palo Alto, CA, USA), and quantified using Qubit 2.0 fluorimeter (Invitrogen, Carlsbad, CA) before loading, then multiplexed in equal molar mass. The pooled DNA libraries were loaded on the Illumina MiSeq instrument according to manufacturer’s instructions. The samples were sequenced using a 2× 250 paired-end (PE) configuration. Image analysis and base calling were conducted by the Illumina Control Software on the Illumina instrument by Genewiz.

### ﻿Data analysis

During pre-processing, raw pair-end sequences were merged using PEAR (Zhang et al. 2014), and cutadapt (Martin 2011) was used for trimming and removing adapter sequences (and later tag sequences). Tags were used to de-multiplex the sequences. In order to reduce the number of sequences, they were clustered within their tag-group using VSEARCH (Rognes et al. 2016) with 99% sequence similarity threshold. Taxonomic classification of sequences was performed using PROTAX (Abarenkov et al. 2018) based on the resulting centroid sequences from VSEARCH. When calculating abundances of different taxa, the cluster sizes were taken into account. Identification of species was performed using UNITE database (https://unite.ut.ee/) version 7.1. We would like to note that the UNITE database, used in our metabarcoding-based species identification, is updated periodically and its potential to resolve species ID is expected to improve each year.

PROTAX-fungi is a tool for taxonomic placement of ITS sequences implemented into the PlutoF platform of the UNITE database for molecular identification of fungi. This tool is able to perform statistically reliable identifications of fungi in spite of the incompleteness of extant reference sequence databases and unresolved taxonomic relationships (Abarenkov et al. 2018). In our study, sample-taxon tables were produced for both the species and genus level of taxonomy. Abundances were counted as the number of sequences whose taxon membership probability exceeded a given threshold of 0.9 to serve as a strict determination of which taxa were included. The choice of 0.9 in generating these tables was selected to serve as a conservative way of determining taxonomic resolution across samples in terms of species and genus identity. This threshold, generally speaking, could be lowered depending on future study designs and available data quality. Here, the estimates of abundance for each taxa generated with this 0.9 threshold were used for subsequent statistical analyses.

The resultant list of species was published at GBIF.orghttps://doi.org/10.15468/r7rxf6 (Shumskaya et al. 2022).

For the statistical analysis, the “species” identified as SynMock taxa were removed from the total list of identified species. Also, several species were identified in sterilized pins sample 0 (Table 1): *Candidaalbicans*, *Candidazeylanoides*, *Wallemiatropicalis*, which could be explained by contamination; these species were removed from all samples before statistical analysis.

Statistical analysis of the occurrence dataset was performed in R (R Core Team 2020) on a filtered version of the dataset without the species found in the negative control or that were not found in more than ten percent of samples. The dataset was then checked for multivariate normality using Mardia’s test in the MVN package (Korkmaz et al. 2014) prior to omnibus analyses. A non-parametric multivariate analysis of variance (PERMANOVA) was then used to determine if fungal community composition varied either based on pin preservation method (dry vs. frozen) or time in days (14, 28, 42, 77, 160). Following omnibus testing, non-metric multidimensional scaling (NMDS) was used to evaluate differences between treatments and time points. We also used a Mann-Whitney (non-parametric t-test) and Kruskal-Wallis (non-parametric ANOVA) to evaluate potential differences between calculated diversity measures for preservation methods and over time. Both the PERMANOVA and NMDS were performed using vegan package for R (Dixon 2003; Oksanen et al. 2020) with all other significance tests for diversity measures being performed in base R. To determine any relevant change to indicator taxa across time an indicator taxa analysis was performed at the genus level following previous multivariate comparisons using the labdsv package (Roberts 2019). To compare annotations for fungal species ecology we retrieved available ecological roles and guild data for each species and its corresponding genus from the FUNguild database (Nguyen et al. 2016) using the fungarium package (Simpson and Schilling 2021). Species and genera with annotations in FUNguild were used for a comparison of both trophic mode and guild annotations using PERMANOVA with reciprocal Kruskal-Wallis tests to evaluate significant differences in fungal roles observed across time.

## ﻿Results

DNA isolated from all collected pins (Table 1) was amplified using PCR with tagged primers (Suppl. material 1: table S1). All PCR amplification results were verified using gel electrophoresis (see Suppl. material 1: fig. S1 for an example). SynMock served as a positive control for both successful PCR reaction settings (Suppl. material 1: fig. S1, lane 5) and high-throughput sequencing runs. Negative control, amplified with the assigned tagged primers, never showed on a gel electrophoresis, or in sequencing or data analysis, confirming no contamination was introduced at the PCR step.

After processing of the high-throughput sequencing results of the PCR amplicons, the subsequent dataset was presented by 67 species for statistical analysis in R. For the statistical analysis, the “species” identified as SynMock taxa were removed from the total list of identified species.

In evaluating the effectiveness of the MycoPins method we used a series of multivariate analyses to determine if 1) a significant effect exists between pin preservation method (dry vs. frozen) and if 2) the method is capable of distinguishing differences that emerge in the fungal community over time (days since placement). We observed in our PERMANOVA results that there was no significant difference in community composition between our two preservation methods (p > 0.05) while there were significant differences in communities over time (F_(4,14)_ = 1.7337, p = 0.036) with no detectable interaction between time and preservation type (p > 0.05).

**Figure 3.**
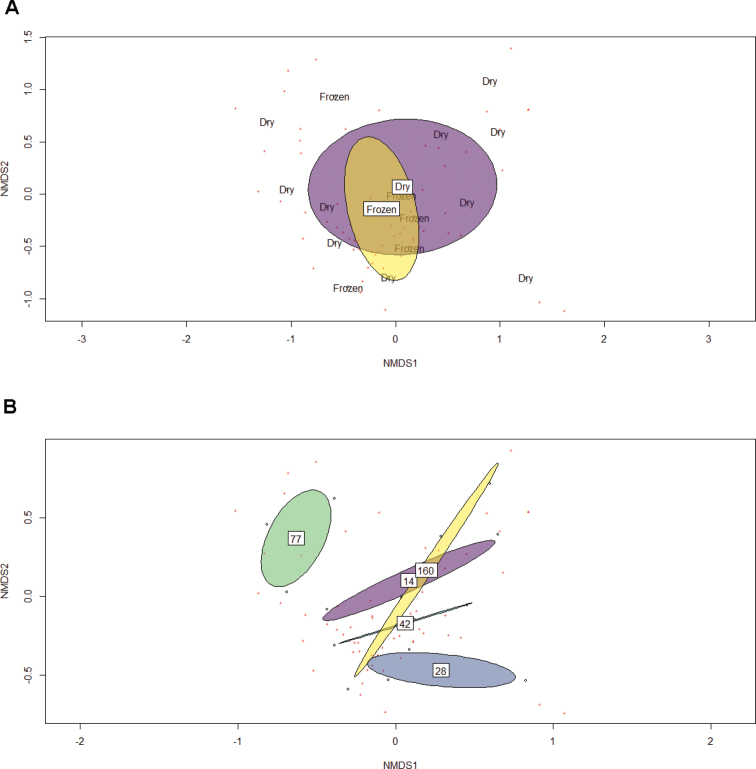
Non-metric multidimensional scaling plots for fungal communities sampled using MycoPins. Ellipses represent 95% confidence intervals with labels for pin preservation method or time elapsed from placement in the center **A**NMDS of the fungal communities observed in pins across 160 days clustered by pin storage method **B**NMDS of fungal communities observed in pins clustered according to date DNA was extracted from placement, numbers: days after inoculation.

The results from the PERMANOVA can be visualized in the NMDS plots (Fig. 3) which show the fungal community clustered both by preservation type (Fig. 3A) and time in days elapsed from pin placement (Fig. 3B). The results of the NMDS support the PERMANOVA showing strong clustering overlap for the two preservation methods (Fig. 3A) and segregation between clusters for the time points sampled (Fig. 3B) which aligns with the findings from the omnibus test.

Through the lens of time elapsed from placement, the NMDS support that the MycoPins method resolved differences in the fungal community over the 160 days in the sampling period despite them being in the same environment. The confidence interval orientation for the time points sampled also shows more similarity for sampling times which are closer together with early fungal communities resembling each other with a slight gradient moving from early time points (upper right) to later time points (bottom left) emerging in the NMDS results.

**Figure 4.**
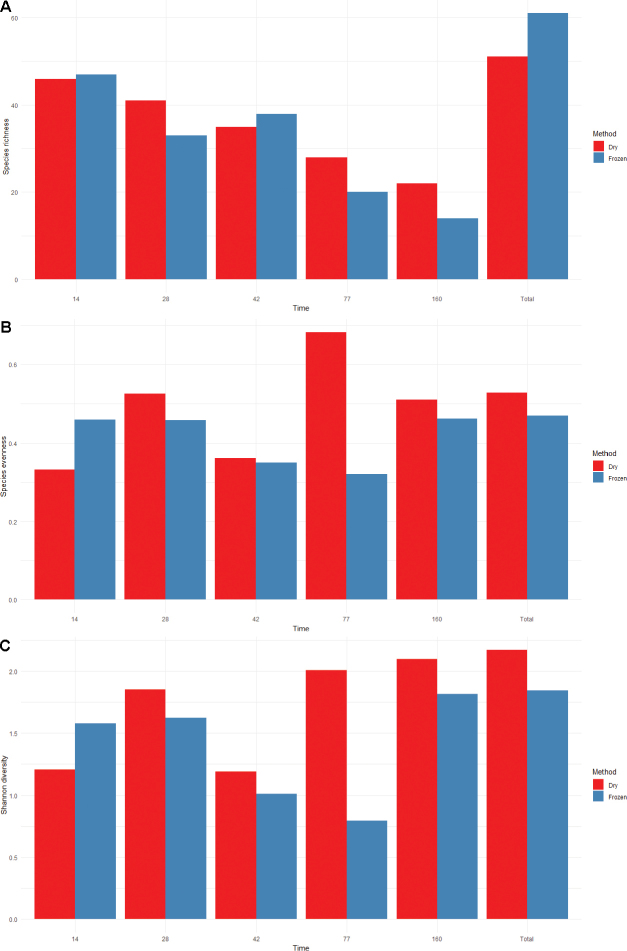
Species richness (**A**), species evenness (**B**), and Shannon diversity (**C**) for both dry (red) and frozen (blue) pins for each time point as well as total values. Time: days from the pin placement.

**Figure 5.**
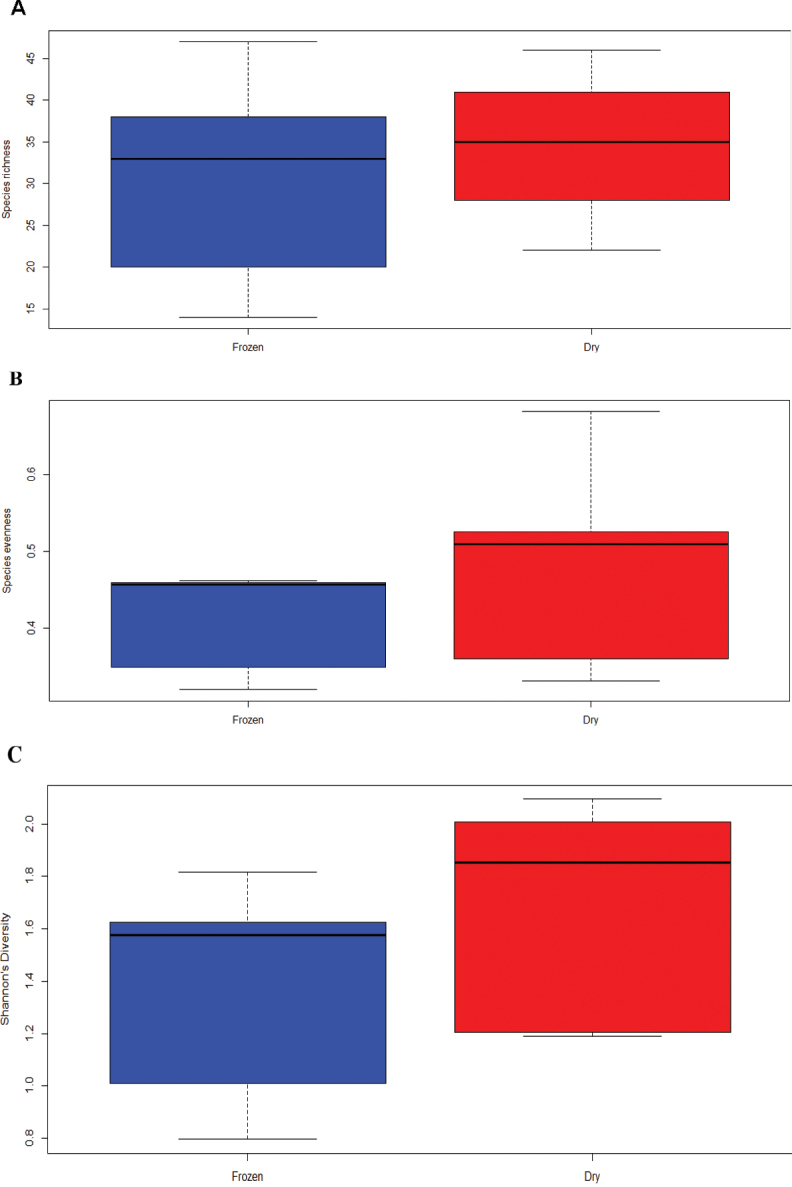
Box plots for values of species richness (**A**), species evenness (**B**), and Shannon’s diversity index (**C**) between our two preservation methods. Box plots: middle line, median; box, interquartile range; whiskers, 5^th^ and 95^th^ percentiles.

**Figure 6.**
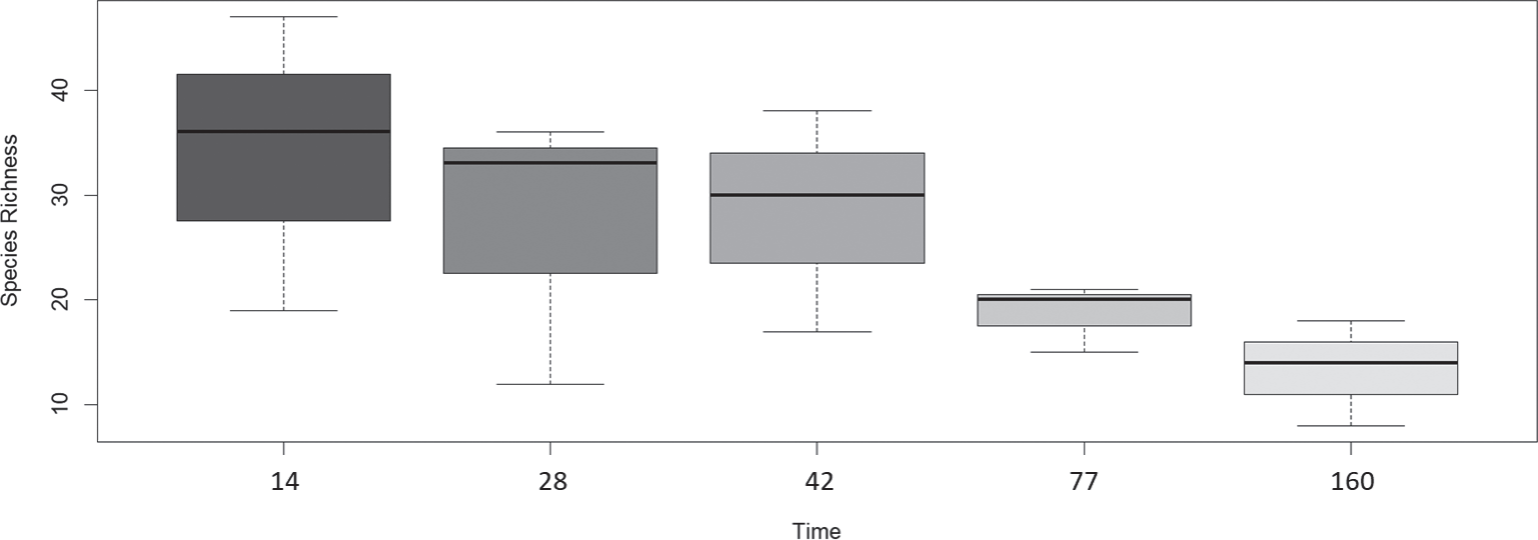
Box plot for values of species richness over time. Box plots: middle line, median; box, interquartile range; whiskers, 5^th^ and 95^th^ percentiles. Time: days after the pin placement.

We have also evaluated trends in measures of species richness (Fig. 4A), evenness (if the sample had a good representation of one species or was dominated by a few, Fig. 4B), and Shannon’s diversity (index usually reported for comparing # of taxa, Fig. 4C). We also compared these diversity measures using significance tests and observed no significant differences between any of our diversity measures based on preservation method (p > 0.05, Fig. 5). Our Kruskal-Wallis test also did not return detectable significant differences between the time points for any of our diversity measures (p > 0.05), though there is a notable visual trend downward in species richness over time (Fig. 6).

**Figure 7.**
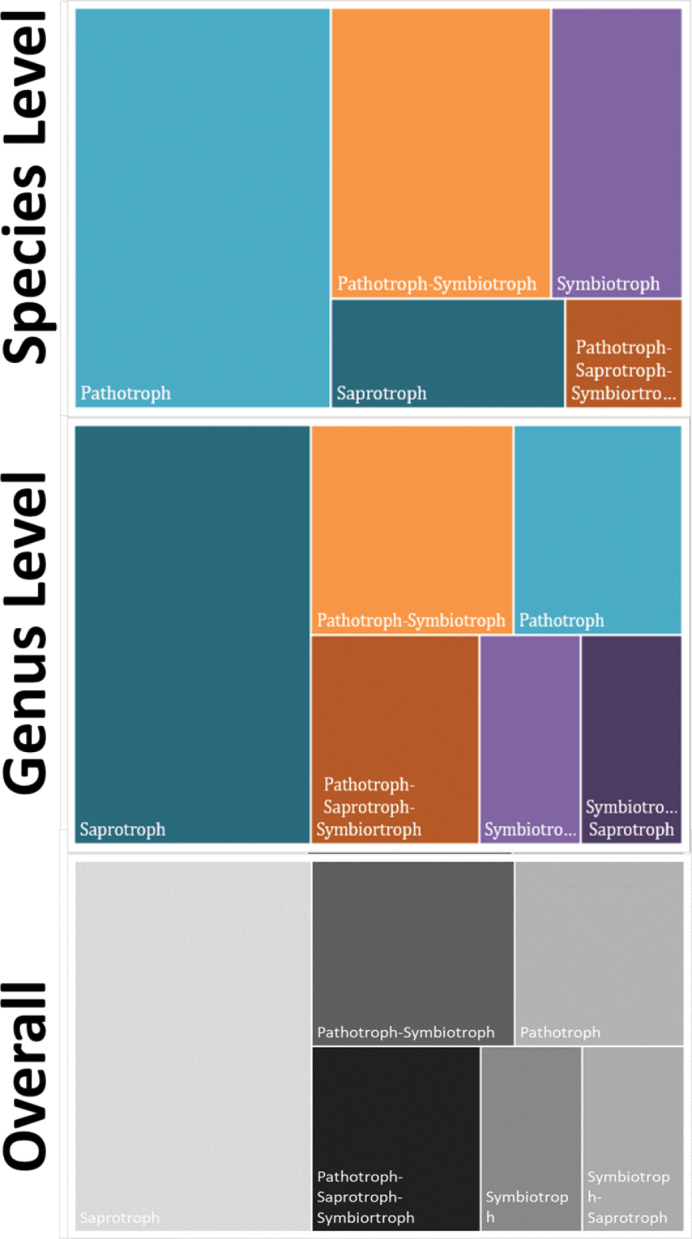
Relative proportion of fungal species, genera and overall fungal taxa (genus or species) identified in the experiment using MycoPins method, with ecological function annotations in FUNguild. Larger segments of the treemap indicate higher numbers of species within an ecological group. Included annotations for species and genus level show feeding modalities, while the overall panel shows observed proportions of ecological guilds.

To evaluate our hypothesis that the MycoPins method can be effectively used to sample saproxylic fungal taxa we retrieved annotations for ecological roles and guilds at the species and genus level. Summaries of available ecological role data at the species level (32% assigned), genus level (54% assigned) and total taxa are presented on Fig. 7. Most represented groups were discovered to be saprotrophic and pathotrophic fungal taxa. Functional guild data for taxa annotated on FUNguild used to evaluate common guilds detected with the MycoPins method highlight the ability of the MycoPins method to detect many plant pathogens, saproxylic, and litter associated taxa. We failed to observe significant differences in our PERMANOVA evaluating trophic mode overall or in guild type (p > 0.05) when considering the data collected by stage overall and reciprocal follow-up tests also were non-significant for trophic groups and guilds when considered in isolation.

To characterize genera that may have indicated changes in the fungal community over time we determined significant indicator genera at each time step and reviewed if they have been previously found to be saproxylic. Table 2 highlights that all of the taxa observed to indicate progression (changes over time) in the fungal community have been observed to be active in wood decomposition or activity in litter layer cellulosic matter decomposition.

**Table 2.** Indicator genera for time points evaluated in the present study. Indicator values were calculated with subsequent significance tests.

Genus	Saproxylic	Litter Saprotroph	Time Step indicated	Indicator Value	p-value
***Taphrina***	X		1	0.748503	0.034
***Sporobolomyces***		X	1	0.936306	0.014
***Myrmecridium***		X	3	0.75	0.037
***Doratomyces***	X		4	0.899293	0.014
***Trichosporon***	X		4	0.92941	0.009
***Diplodia***		X	5	0.962963	0.018
***Valsa***	X		5	1	0.008
***Puccinia***	X		5	0.867925	0.012

## ﻿Discussion

Here we suggest a pin-based monitoring method of fungal colonization as a new protocol of biodiversity exploration that is not yet widely utilized. The method allows monitoring of fungal colonization in various biotopes. In effect, pins act as standardized traps and colonizable targets mimicking with some limitations natural inputs of fine woody debris: despite the convenience of their use, pins are still a semi-artificial, processed, autoclaved, no-bark artifacts, dried before placement. Sterile (autoclaved) wood, obviously, does not occur in nature, but to study colonization process we believe it is more important and more cost efficient to use autoclaved wood rather than account for resident fungi (which would dramatically increase processing time and cost). At the same time, wood is natural material, which can only be standardized to a certain limit – while choice of tree species and wood treatment improve comparability, differences in wood density (fast vs. slow wood) across pins remain beyond our full control; however, pins in the same batch are typically very uniform. We can recommend for the future studies to document or to unify ranges of tree rings per pin. We emphasize that the suggested method is not designed to attempt an exhaustive sampling of fungal communities *in situ*, hence, there is no need to replace an alternative direct sampling of soil or fine woody debris for the metabarcoding based surveys. Instead, using the sterile wood pins, we offer an opportunity to uncover still mysterious processes of fungal colonization of wood. There is a great deal of flexibility for study designs with the pins placement patterns and with removal frequencies for the field experiments resulting in the time series data; direct sampling of the environment can be combined with pin-based studies.

The outlined protocol used in our test dataset demonstrate that the pipeline suggested is capable of resolving differences in community assembly over time for fungal species monitoring.

Our study was inspired by the Global Teabag index study (Keuskamp et al. 2013), but shifted the focus of the approach from loss of biomass to colonization processes. The method was capable of resolving fungal community differences effectively using either dried or frozen pins. When comparing dried and frozen pins, pins collected at the same time point are more similar in fungal community composition than pins of the same preservation type at other time points. This likely means that, while freezing or drying may capture different rare taxa, researchers do not need to have access to a freezer to store samples if in the field to generate an imprint of the common taxa. The significant difference in the overall fungal community composition observed in our omnibus test between pins collected at different times, on the other hand, indicates that this method is likely well suited to evaluating how fungal communities change over time and are distributed based on community assembly. Trends observed in the diversity measures presented above highlight a gradual reduction in species richness over time (Fig. 6), though a relatively consistent evenness and diversity, potentially suggesting succession favoring a set of species being recruited in the progression of decay.

Beyond the ability of the MycoPins method to detect changes in the fungal community over time, we were also able to use it to detect changes in relevant ecological functional groups and guilds. While there are a wide range of fungal types within our sampled pins (e.g. endophytes, pathogens), suggesting that this method could be broadly used for fungal community sampling, we found support for our hypothesis that wood saprobes/saproxylic taxa colonize sterile furniture pins (Hypothesis 1a). Our approach allows for flexibility depending on observed taxa when considering functional roles by reviewing widely available annotations in databases such as FUNguild. As mentioned above, however, annotations for many taxa in our sample data were missing and manual research for annotations for individual taxa may not be tenable as an option for many researchers wishing to employ our method. We explored an alternate approach for monitoring specific taxa that contribute to wood decay that we suggest for researchers who also face annotation issues when considering functional roles in the form of our presented use of indicator values. As seen in Table 2, our method was able to detect significantly active indicator genera at four out of five of our time stages (with time stage two not having significantly high indicator values and therefore no indicator species). Subsequent manual annotation and research demonstrated that many of the taxa which have significant indicator values also have been shown to be saproxylic or contribute to the overall process of wood decay with the later time points having increasing numbers of taxa involved in wood decay compared to earlier stages. This shows that, despite cases like ours where existing database annotation may be a limitation, this method can still be employed to monitor potential changes in saproxylic species without placing unreasonable time burdens on researchers.

A tradeoff of the proposed method to study colonization processes is that pins are exposed to natural communities but the method is blind to priority effects, which is known to determine further colonization of the wood (Hiscox et al. 2015). A more elaborate study setup, still in natural habitats, may include use of pins precolonized by target species, (e.g. those common at the study site at the early colonization stages); such a workflow would start from i) the morphological or DNA survey of the pioneer species, ii) precolonization of pins with these species, see method developed by Schigel and Oivanen for species reintroduction (Abrego et al. 2016), and iii) colonization studies as presented here. To maximize designs that allow for comparison with natural forest communities, we recommend researchers order pins from sawmills that use locally and sustainably harvested timber in their study area, and that pins be made from the same tree species as at the sampling sites. As timber trade and timber certification is not always transparent and trustworthy, partnering with a local sawmill, even if more costly than using mass market pins, is preferred.

We were also able to support the hypothesis that the preservation method used by researchers will not significantly change the observations made by researchers using the MycoPins method (Hypothesis 2). Overall trends for species composition in our omnibus test and NMDS (Fig. 3B) find a high degree of similarity for fungal community composition observed using the two methods. In addition, trends observed for our diversity measures show that similar trends are captured using either dried or frozen pins such as the overall decline over time for species richness observed using both preservation methods (Fig. 4A) and all three diversity measures lack detectably significant differences. We conclude, overall, that the two preservation methods can be used depending on researcher preference and available equipment, though slightly higher (though non-significant) overall community richness in frozen pins, as well as better DNA preservation over time, lead us to suggest freezing when possible if DNA extraction and handling is considerably delayed after the pins collection. Many fungal taxa are also known to be active in plant decomposition or to be plant pathogens with the potential that they may be opportunistically contributing to wood decomposition. Given that our knowledge of fungal functional group and guild are still developing (many taxa we identified lack any annotation) coupled with the fact that many taxa known to be endophytic were found within the pins, it seems likely that our method can expand our knowledge of previously unknown saproxylic species as well as increasing resolution on global distribution for those already known.

## Supplementary materials

Supplementary material 1 Sequences of the primers used in the experiment and ITS2 fragment amplified from DNA extracted from saw dust of the pins Data type docx file https://binary.pensoft.net/file/827823This dataset is made available under the Open Database License (http://opendatacommons.org/licenses/odbl/1.0/). The Open Database License (ODbL) is a license agreement intended to allow users to freely share, modify, and use this Dataset while maintaining this same freedom for others, provided that the original source and author(s) are credited.Maria Shumskaya, Nicholas Lorusso, Urvi Patel, Madison Leigh, Panu Somervuo, Dmitry Schigel
