# Supplementary material for: ﻿MycoPins: a metabarcoding-based method to monitor fungal colonization of fine woody debris
Source: MycoKeys. 2023 Mar 21;96:77–95. doi: 10.3897/mycokeys.96.101033 (PMC10196935; doi:10.3897/mycokeys.96.101033)
Supplement: Supplementary material 1 — Sequences of the primers used in the experiment and ITS2 fragment amplified from DNA extracted from saw dust of the pins [file mycokeys-96-077-s001.docx]

**SUPPLEMENTARY DATA**

**MycoPins: a metabarcoding-based method to monitor fungal colonization of fine woody debris**

M. Shumskaya^1^, N. Lorusso^1,2^, U. Patel^1^, M. Leigh^1^, P. Somervuo^2^, D. Schigel^3^

1 – Kean University, USA, 2 – University of North Texas at Dallas, USA, 3 - Biological and Environmental Sciences, University of Helsinki, Finland

MycoKeys, 2023

Supplementary Table S1. Sequences of the primers used in the experiment (based on Clemmensen, 2016)

Schematic primer structure (fITS7 used as an example):

5’ -C -N N N N N N N N -T- G T G A R T C A T C G A R T C T T T G-3’

Ligation site (always C)

Ligation site (always C)

Ligation site (always C)

Ligation site (always C)

tag

tag

tag

tag

Linker (always T)

Linker (always T)

Linker (always T)

Linker (always T)

Primer fITS7 binds to template

Figure 5. Scheme of the MycoPins method pipelinePrimer fITS7 binds to template

Figure 6. Scheme of the MycoPins method pipeline

Figure 7Figure 8. Scheme of the MycoPins method pipelinePrimer fITS7 binds to template

Figure 9. Scheme of the MycoPins method pipelinePrimer fITS7 binds to template

| Primer pair # | Primer Name | Primer Sequence, from 5' |
| --- | --- | --- |
| 1 | tag_1F | CACACGATCT GTGARTCATCGAATCTTTG |
|  | tag_1R | CACACGCTGT TCCTCCGCTTATTGATATGC |
| 2 | tag_4F | CACATAGTCT GTGARTCATCGAATCTTTG |
|  | tag_4R | CACATGTCGT TCCTCCGCTTATTGATATGC |
| 3 | tag_5F | CACATGACTT GTGARTCATCGAATCTTTG |
|  | tag_5R | CACGCAGCAT TCCTCCGCTTATTGATATGC |
| 4 | tag_6F | CACGATCAGT GTGARTCATCGAATCTTTG |
|  | tag_6R | CACTAGCGCT TCCTCCGCTTATTGATATGC |
| 5 | tag_7F | CACGTGCTCT GTGARTCATCGAATCTTTG |
|  | tag_7R | CACTATGCAT TCCTCCGCTTATTGATATGC |
| 6 | tag_8F | CACTATAGCT GTGARTCATCGAATCTTTG |
|  | tag_8R | CACTCACACT TCCTCCGCTTATTGATATGC |
| 7 | tag_9F | CACTATGTGT GTGARTCATCGAATCTTTG |
|  | tag_9R | CACTCTGAGT TCCTCCGCTTATTGATATGC |
| 8 | tag_10F | CACTCAGAGT GTGARTCATCGAATCTTTG |
|  | tag_10R | CACTGATCAT TCCTCCGCTTATTGATATGC |
| 9 | tag_11F | CACTCTCACT GTGARTCATCGAATCTTTG |
|  | tag_11R | CACTGTATGT TCCTCCGCTTATTGATATGC |
| 10 | tag_12F | CACTGCTACT GTGARTCATCGAATCTTTG |
|  | tag_12R | CAGACATAGT TCCTCCGCTTATTGATATGC |


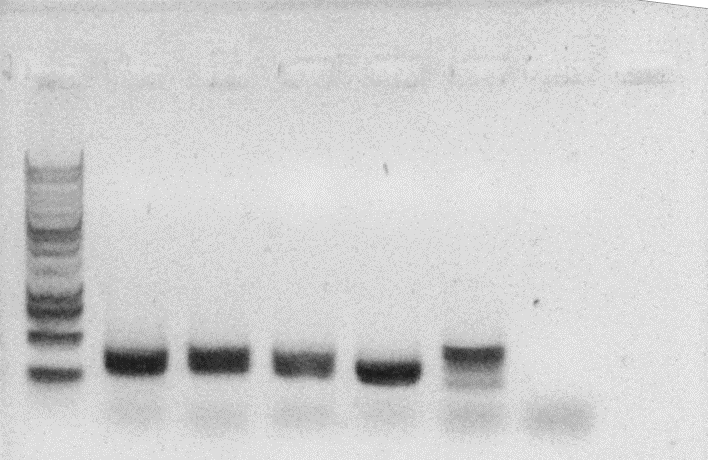


M 1 2 3 4 5 6

Figure 10M 1 2 3 4 5 6

Figure 11. Non-metric multidimensional scaling plots for fungal communities sampled using MycoPins. Ellipses represent 95% confidence intervals with labels for pin preservation method or time elapsed from placement in the center. A) NMDS of the fungal communities observed in pins across 160 days clustered by pin storage method. B) NMDS of fungal communities observed in pins clustered according to date DNA was extracted from placement, numbers: days after inoculation.

Figure 12M 1 2 3 4 5 6

Figure 13M 1 2 3 4 5 6

1000

500

250

Supplementary Figure S1. ITS2 fragment amplified from DNA extracted from saw dust of the pins (selected samples are representative for all studied pins).

Sample numbers refer to Table 1.

M -1 kb DNA ladder (bp, Promega) ,

1 – sample #1, frozen pin, primers tag_4F/R,

2 – sample #2, frozen pin, primers tag_5F/R,

3 – sample #3, frozen pin, primers tag_7F/R,

4 – sample #4, frozen pin, primers tag_8F/R,

5 – SynMock community, primers tag_10F/R,

6 – negative control, primers tag_9F/R
